# Engineered Fluorescent Variants of Lactadherin C2 Domain for Phosphatidylserine Detection in Flow Cytometry

**DOI:** 10.3390/biom15050673

**Published:** 2025-05-06

**Authors:** Ekaterina Koltsova, Albina Avilova, Elena Nikolaeva, Nikita Kolchin, Kirill Butov

**Affiliations:** 1 Dmitry Rogachev National Medical Research Center of Pediatric Hematology, Oncology and Immunology, Moscow 117997, Russiakrbutov@gmail.com (K.B.); 2 Center of Theoretical Problems of Physico-Chemical Pharmacology of the Russian Academy of Sciences, Moscow 109029, Russia; 3 Department of Molecular Biology and Medical Biotechnology, Pirogov Russian National Research Medical University, Moscow 117513, Russia

**Keywords:** lactadherin, annexin V, phosphatidylserine, extracellular vesicles, platelets, microvesicles, apoptosis

## Abstract

Phosphatidylserine (PS) is an essential phospholipid and an emerging biomarker involved in key biological processes. While annexin V (axV) is the most widely used tool for PS detection, its calcium-dependent binding and other limitations have spurred interest in alternative probes. The lactadherin C2 domain (lactC2) offers a promising alternative, addressing many of the drawbacks associated with axV. However, its broader adoption has been hindered by challenges in production and modification for convenient experimental use. Here, we demonstrate the successful in-house engineering of fully functional recombinant bovine lactC2-based fluorescent sensors and compare their key parameters to axV probes. We show that mNeonGreen–lactC2 fusion exhibits calcium-independent binding with a comparable dissociation constant for 20% PS liposomes. We also demonstrate the detrimental effects of primary amine modification on lactC2’s PS binding efficiency, suggesting the preferential use of fluorescent protein fusion or alternative approaches. Finally, we show that unlike full-length lactadherin or axV, lactC2 inhibited thrombin generation only at high concentrations (>250 nM) in coagulation assays. These findings establish recombinant lactC2 as a versatile and promising PS sensor, with potential applications in experimental settings where axV might be unsuitable

## 1. Introduction

Phosphatidylserine (PS), an anionic phospholipid, is a minor yet vital component of biological membranes, typically representing up to 10 mol% of the total phospholipid content in mammalian cells [1]. Despite its limited presence, PS plays a significant role in cellular physiology. Under healthy conditions, PS is asymmetrically concentrated in the inner leaflet of the plasma membrane, maintaining a distinct distribution [2]. The loss of this asymmetry, resulting in PS exposure on the outer membrane surface, is a key event in apoptosis, facilitating the recognition and removal of dying cells [3]. Beyond apoptosis, PS externalization is essential for a variety of critical biological processes. These include blood clotting, bone formation, muscle cell fusion, egg–sperm interaction, and photoreceptor renewal [4,5,6,7]. In addition to its exposure on the surfaces of cells, phosphatidylserine is also a universally recognized marker of extracellular vesicles, used for their detection in biological samples. The universality of this marker is explained by the loss of lipid asymmetry during vesicle shedding [8].

Measurements of PS exposure are widely used in membrane-related biochemical and biophysical studies. One of the many clinically relevant applications fields of study is in describing coagulation factor assembly on lipid membranes [9].

Annexin V is currently the standard marker for PS on the surfaces of cells and extracellular vesicles. However, this marker is not without certain limitations, which has prompted researchers to explore alternative molecules based on protein sequences containing phosphatidylserine-binding domains, such as lactadherin [10], evectin-2 [11], and Tim4 [12], with lactadherin being the most extensively studied among them. The research group of Gilbert et al. [10] was the first to isolate full-length lactadherin from bovine milk. Lactadherin differs from annexin V in its stronger affinity for membranes with increased curvature [13], as well as its ability to successfully bind to membranes containing less than 4% PS [14,15]. Additionally, lactadherin binding does not depend on the presence of phosphatidylethanolamine in the membrane [15] or the presence of calcium ions in the solution [16]. Importantly, the binding of annexin V leads to its oligomerization and the formation of a lattice on the membrane surface [17,18], making it unsuitable for use in combination with other lipid markers due to steric interactions with them. In contrast, the binding pattern of lactadherin to PS leaves the membrane surface accessible for other lipid markers. Overall, this makes lactadherin a very promising PS probe in experimental settings where axV may produce artificial results. Currently, full-length lactadherin is successfully used for the detection of procoagulant platelets alongside annexin V [19,20]. However, the need for a labor-intensive procedure to isolate the full-length protein has slowed its widespread adoption as a new and universal PS marker.

Lactadherin binds to phosphatidylserine (PS) through its C2 domain, which shares structural homology with the discoidin family of C-domains, particularly the lipid-binding regions of coagulation factors V and VIII [10]. This evolutionary relationship suggests that the recombinant production of the isolated C2 domain could circumvent the challenges of purifying full-length lactadherin. While such C2 domain-based probes have been developed and described in the literature [21,22], their application has been largely restricted to genetically encoded intracellular PS detection.

Although several studies have successfully expressed the lactadherin C2 domain in *E. coli* [16,23,24], practical limitations persist. Most notably, the tendency of the recombinant protein to precipitate during purification [21] has hindered its broader adoption as a research tool. This is particularly unfortunate given flow cytometry’s central role in modern cell and extracellular vesicle research, where its capacity for high-throughput, multiparametric, single-particle analysis makes it ideally suited for PS detection. However, despite the growing interest in lactadherin and its isolated C2 domain as PS markers with unique advantages over annexin V, such probes are still rarely used in the current literature.

In addition to its potential as a phosphatidylserine sensor suitable for experimental applications, full-length lactadherin has been shown to potentially serve as a phospholipid-blocking anticoagulant. This is due to its ability to compete with coagulation factors V and VIII for the binding sites, thereby inhibiting the prothrombinase and intrinsic tenase complexes [10]. The isolated C2 domain of lactadherin remains unexplored in terms of its effects on coagulation, which limits its use in experiments involving the assembly of active coagulation factor complexes.

The aims of the current study were to develop a range of fluorescent recombinant sensors based on the lactadherin C2 domain, evaluate their suitability for use in the flow cytometry of cells and extracellular vesicles, and estimate their binding affinity. Additionally, we evaluated the isolated C2 domain’s effects on blood coagulation, as this sensor shows significant potential for thrombosis and hemostasis research. For the biophysical characterization, we fused the lactadherin C2 domain with one of the brightest monomeric fluorescent proteins, mNeonGreen [25]. To expand the range of available probes, we also describe the perfomance of TagBFP fusion and primary amine-conjugated variants. As for the effect on the coagulation, we report that the isolated lactadherin C2 domain inhibits thrombin generation in platelet-free plasma using one of the most common experimental systems, although only at high concentrations (above 250 nM).

## 2. Materials and Methods

### 2.1. Expression Vector Construction

The coding sequence of the bovine lactadherin C2 domain (lactC2, residues 270–427) was obtained from the Lact-C2-GFP plasmid [22] (the Lact-C2-GFP was a gift from Sergio Grinstein, Addgene plasmid #22852; http://n2t.net/addgene:22852 (assessed on 27 March 2025); RRID:Addgene_22852). The codon-optimized (*Homo sapiens*) sequence of mNeonGreen (mNG) was obtained from the pmNeonGreenHO-G plasmid [26] (the pmNeonGreenHO-G was a gift from Isei Tanida, Addgene plasmid #127912; http://n2t.net/addgene:127912 (assessed on 27 March 2025); RRID:Addgene_127912). The coding sequence of TagBFP was obtained from the pTagBFP-N plasmid (Evrogen, Moscow, Russia) [27]. The coding sequence of human annexin V (axV) was obtained from the pProEx.Htb.annexin V plasmid [28].

A polymerase chain reaction was performed to insert open reading frames using Q5 High-Fidelity Polymerase (NEB) according to the manufacturer’s instructions. For the insertion of lactC2 and mNG sequences, restriction sites were introduced as 5′ primer overhangs. Subsequently, the protein-encoding fragments were subcloned into the pET-28a(+) vector (Novagen, Darmstadt, Germany) using T4 ligase (NEB, Ipswich, MA, USA). The TagBFP and axV sequences were subcloned into pET-28a(+) by the ClonExpress Ultra One-Step Cloning Kit (Vazyme, Nanjing, China).

The resulting protein sequences contained either N-terminal mNG or TagBFP and C-terminal lactC2, N-terminal axV, and C-terminal mNG, or isolated lactC2 or axV sequences. To obtain the hydrophobic spike 1 and 3 mutant of lactC2 with abolished membrane binding [16], point mutations resulting in W26A/G27A/L28A and F81A/G82A replacements in the lactC2 sequence (lactC2 mut) were introduced via site-directed mutagenesis. All protein sequences included an N-terminal 6xHis-tag. The obtained plasmids were propagated in DH5α *E. coli* cells. Plasmid clones were isolated using the HiPure Plasmid Mini Kit (Magen, Guangzhou, China). Correct final sequences were confirmed via Sanger sequencing using T7 forward and T7 terminal primers. The primer sequences used in this study are provided in Table S1 in Supplementary Materials.

### 2.2. Protein Synthesis and Purification

The *E. coli* strain Rosetta-Gami 2(DE3)pLysS (Novagen, Darmstadt, Germany) was transformed with pET-28a(+)-containing sequences for mNG-lactC2, TagBFP-lactC2, mNG-lactC2 mut, axV-mNG, lactC2, or axV, and glycerol stocks were prepared. For protein synthesis, 50 mL in a baffled 125 mL Erlenmeyer flask (NEST, Wuxi, China) of Terrific Broth medium containing 0.5% *v/v* glycerol, 50 µg/mL kanamycin, and 34 µg/mL chloramphenicol was inoculated with 500 µL of overnight culture inoculated from glycerol stock. The culture was grown at 37 °C until the OD600 range reached 1.3–1.6, induced with 0.1 mM of IPTG, and then expressed for 12 h at 18 °C in an orbital shaker at 195 RPM with a 50 mm orbit (Senova Biotech, Shenzhen, China). The biomass was harvested via centrifugation at 5000× *g* for 10 min. The pellet was lysed using a lysis buffer containing 20 mM of Tris-HCl, 300 mM of NaCl, 10 mM of imidazole, cOmplete Protease Inhibitor Cocktail (Roche, Basel, Switzerland), and 0.1% Triton X-100, followed by additional disruption via sonication (15 impulses, 25% amplitude, 10 s per impulse followed by 30 s rest on ice). The crude lysate was clarified via centrifugation at 8000× *g* at 4 °C for 30 min.

Protein isolation was performed using gravity columns packed with 500 µL of Ni-NTA Protino resin (Macherey-Nagel, Duren, Germany), according to the manufacturer’s instructions. The proteins were eluted in three fractions of 500 µL each. The results of the Ni-NTA chromatography were confirmed via SDS-PAGE. The protein-containing fractions were pooled and buffer-exchanged using gravity columns packed with Smartdex G-25 fine resin (Smart-Lifesciences, Guangzhou, China) into a storage buffer containing 20 mM of HEPES, 150 mM of NaCl, and 100 mM of trehalose (pH 7.4). The solution was filtered through a 0.2 µm PES syringe filter (NEST, China) and immediately frozen at −80 °C at a concentration of approximately 1 mg/mL. Filtration prior to freezing and the addition of 100 mM of trehalose to the storage buffer were performed to prevent the observed tendency of lactC2 to form aggregates.

### 2.3. Assessment of LactC2-Based Sensor Solubility via Dot Blot

For the low-volume preparative synthesis, 4 mL of LB Lennox medium supplemented with 0.5% (*v*/*v*) glycerol, 50 µg/mL of kanamycin, and 34 µg/mL of chloramphenicol in 50 mL Falcon-type bioreactor tubes was inoculated with 40 µL of glycerol stock containing the *E. coli* strain Rosetta-Gami 2(DE3)pLysS (Novagen, Darmstadt, Germany) expressing lactC2, mNG-lactC2, or TagBFP-lactC2. The culture was grown at 37 °C until the OD600 reached 0.7, induced with 0.1 mM of IPTG, and then expressed for 12 h at 18 °C in an orbital shaker at 195 RPM with a 50 mm orbit (Senova Biotech, China). The biomass from 1.5 mL of the culture was harvested by centrifugation at 9000× *g* for 5 min, yielding pellets weighing 33–34 mg. The pellets were lysed using 1.5 mL of lysis buffer (20 mM Tris-HCl, 300 mM NaCl, 10 mM imidazole, cOmplete Protease Inhibitor Cocktail (Roche, Switzerland) and further disrupted via sonication (7 pulses at 25% amplitude, 10 s per pulse with 30 s of rest on ice). The crude lysate was clarified via centrifugation at 12,300× *g* for 3 min.

For the dot blot analysis, 2 µL samples of both crude and clarified lysates, representing the total and soluble protein fractions, were applied to a nitrocellulose membrane with a pore size of 0.45 µm (BioRad, Hercules, CA, USA) in triplicate and dried for 30 min. The membrane was blocked with EveryBlot Blocking Buffer (BioRad, Hercules, CA, USA) and incubated with a primary monoclonal mouse anti-His Tag antibody (clone J099B12, RRID AB_11204427; BioLegend, San Diego, CA, USA). After washing with TBS containing 0.1% Tween 20, the membrane was incubated with a secondary polyclonal HRP-conjugated goat anti-mouse IgG (H + L) antibody (RRID AB_2536527; Invitrogen, Waltham, MA, USA). Signal detection was performed using Clarity Western ECL Substrate (BioRad, CA, USA) on a ChemiDoc MP Imaging System (BioRad, Hercules, CA, USA).

The analysis of the obtained images was performed using the Fiji Protein Array Analyzer plugin [29]. Background subtraction was carried out using the rolling ball algorithm with a 25-pixel radius. The integrated density over the area of the dot was used as the primary measurement. The percentage of protein yield in the soluble fraction was calculated as the ratio of the signal from the clarified lysate spots to the averaged signal from the crude lysate spots.

### 2.4. Preparation and Labeling of Model Membranes

The phospholipid liposomes were prepared using a protocol recommended by Avanti Polar Lipids (Birmingham, AL, USA) with minor modifications [30]. Briefly, chloroform was evaporated from a mixture of L-α-phosphatidylcholine (PC) (Sigma Aldrich, St. Louis, MO, USA) and 1,2-diacyl-sn-glycero-3-phospho-L-serine (PS) (Sigma Aldrich, MO, USA) at a PS/PC ratio of 20:80, in the presence of 0.01 mol% of the DiD lipophilic tracer (Lumiprobe, Moscow, Russia), under a continuous stream of argon. The dry lipid film was hydrated by adding HEPES-buffered saline (20 mM HEPES, 150 mM NaCl, pH 7.4, hereafter referred to as HBS) and agitating the mixture at 60 °C for 1 h. The large multilamellar vesicles formed after hydration were disrupted by several freeze–thaw cycles, followed by extrusion through a 1.0 µm membrane at 60 °C. The liposomes were used fresh, and the storage time at 4 °C prior to experiments did not exceed 24 h.

Activated platelets were prepared as described in [31] with minor modifications. Briefly, whole blood samples collected into 3.8% sodium citrate tubes were centrifuged at 100× *g* for 8 min, and the supernatant (platelet-rich plasma, PRP) was collected. The PRP was mixed with 110 mM of sodium citrate at pH 5.5 (1:3 *vol*:*vol*), and the platelets were separated from the PRP via centrifugation at 400× *g* for 5 min. The pelleted platelets were washed once with HBS, mixed again with 110 mM of sodium citrate at pH 5.5 (1:3 *vol*:*vol*), and centrifuged at 400× *g* for 5 min. The washed platelets were resuspended in HBS containing 2.5 mM of calcium chloride and activated with 1 µM of calcium ionophore A23187 (Sigma Aldrich, MO, USA) for 10 min at room temperature (RT).

The platelet-derived extracellular vesicles (platelet EVs) were collected as described in [32], with minor modifications. Briefly, the washed platelets were activated with 1 µM of calcium ionophore A23187 (Sigma Aldrich, MO, USA) in the presence of 2.5 mM of calcium chloride for 10 min at RT. The suspension was centrifuged at 1850× *g* for 10 min to remove the platelets, and the EV-rich supernatant was harvested for flow cytometry studies.

### 2.5. Evaluation of LactC2-Based Sensors’ Binding Affinity (Equilibrium Binding)

For the binding assay, 20:80 PS/PC liposomes pre-stained with the DiD lipophilic dye were incubated for 1 h with mNG-lactC2 in HBS or axV-mNG in HBS supplemented with CaCl_2_ (2.5 mM). The achievement of binding equilibrium was confirmed by additional fluorescence measurements taken 3 h after the start of incubation (see Figure S1 in the Supplementary Materials). The samples were then analyzed using a NovoCyte flow cytometer (ACEA Biosciences Inc., San Diego, CA, USA) and FlowJo v10.5.3 software (BD Life Sciences, Franklin Lakes, NJ, USA). The threshold boundary for separating liposomes from debris was determined as an APC signal higher than that of the unstained liposomes. The phospholipid liposomes were gated as DiD-positive events beyond this boundary (excitation: 640 nm; band filter: 675/30 nm). The signal from the membrane-bound proteins was registered in the FITC channel (excitation: 488 nm; band filter: 530/30 nm). The liposomes were diluted to achieve approximately 10,000 events per 100 µL of sample. Liposomes labeled with mNG-lactC2 mut were used as a specificity control for mNG-lactC2 binding, while liposomes labeled with axV-mNG in the presence of 10 mM EDTA were used as a specificity control for axV-mNG binding. The vesicular nature of the detected events was confirmed by performing detergent treatment control with 0.1% Triton X-100, which resulted in event disappearance.

To quantify the number of bound protein molecules per liposome, we prepared calibrator platelets as described in [33]. Briefly, washed platelets pre-incubated with 1 µM of calcium ionophore A23187 were incubated with various concentrations of lactC2-mNG and fixed with 2% *v/v* formaldehyde, followed by purification with unreacted dye. The fluorescence level of these calibration platelets was measured using a fluorometer (Eppendorf PlateReader AF2200, Hamburg, Germany) and converted to the number of fluorophore molecules per platelet using a calibration curve. Finally, the calibrator platelets with known numbers of attached dye molecules were used to construct a flow cytometry calibration curve, enabling the conversion of the mean population fluorescence into the number of protein molecules per liposome. The mean binding parameters were calculated from four independent experiments. Fitting was performed using non-linear least squares regression. After subtracting non-specific binding, the binding curves were fitted with a Hill equation:

$$\Theta =\frac{{\left[L\right]}^{h}}{K_{D}+{\left[L\right]}^{h}}=\frac{{\left[L\right]}^{h}}{K_{d}^{h}+{\left[L\right]}^{h}}\;$$

where *Θ* is the fractional occupancy of the binding sites, [*L*] is the total ligand concentration, *K_D_* is the apparent dissociation constant derived from the law of mass action, *K_d_* is the ligand concentration producing half occupation (microscopic dissociation constant), and *h* is the Hill coefficient.

A data analysis was performed using GraphPad Prism 8.0 (GraphPad Software, Boston, MA, USA).

### 2.6. Fluorescence Labeling

Fifty micrograms of purified lactC2 was labeled with AF647 NHS ester (Lumiprobe, Russia) according to the manufacturer’s instructions. Briefly, AF647 was dissolved in high-grade DMSO (ThermoFisher Scientific, Waltham, MA, USA). For protein labeling, 2×, 4×, 8×, 10×, and 20× molar excesses of NHS ester were added to the 50 micrograms of lactC2 in 0.1 M of sodium bicarbonate solution (pH = 8.1) to achieve varying degrees of labeling (DOL) and incubated for 1 h at room temperature. After removing the unbound dye and performing buffer exchange using Amicon Ultra 10kDa centrifugal filters (Merck, Darmstadt, Germany), the labeling efficiency was determined by comparing the absorbance at 280 nm with that at 655 nm, with correction for the absorbance of AF647 at 280 nm using NanoDrop OneC (ThermoFisher Scientific, USA). The labeled lactC2 is referred to hereafter as lactC2-AF647. To account for non-specific background signals in flow cytometry experiments, free AF647 NHS ester was quenched with 50 mM of glycine and used as a negative control. The labeling of axV was performed similarly to lactC2, although only an 8× molar excess of dye to protein was used.

### 2.7. Flow Cytometry Evaluation of LactC2-Based Fluorescent Probes

To evaluate the performance of recombinant lactC2-based sensors as potential fluorescent probes for PS on cells and extracellular vesicles, activated platelets were incubated with 100 nM of mNG-lactC2, TagBFP-lactC2, lactC2-AF647 (with varying degrees of labeling, DOLs), or axV-AF647 for 30 min at room temperature (RT). The platelets were labeled based on FSC and SSC signals. Free AF647, quenched with glycine, was added to the wells at the same molar concentrations as determined for lactC2 labeled with various DOLs.

We also evaluated mNG-lactC2 and TagBFP-lactC2 as PS sensors on platelet EVs. The EV samples were first incubated with 1 µL of an anti-integrin IIb antibody (CD41-APC, clone HIP8) (Elabscience, Wuhan, China) for 30 min at room temperature (RT) in 30 µL samples, followed by dilution to 300 µL to minimize non-specific labeling and binding with 100 nM of mNG-lactC2 or TagBFP-lactC2 at RT for 30 min. The antibody stock was pre-centrifuged for 3 min at 12,300× *g* to minimize the likelihood of antibody aggregates entering the sample. The threshold boundary for separating EVs from debris was determined based on the analysis of unstained EVs. The EVs were gated as APC-positive events beyond this boundary (excitation: 640 nm; band filter: 675/30 nm). The vesicular nature of the detected events was confirmed by performing detergent treatment control with 0.1% Triton X-100, which resulted in event disappearance.

The samples were analyzed using a NovoCyte flow cytometer (ACEA Biosciences Inc., CA, USA) and FlowJo v10.5.3 software (BD Life Sciences, NJ, USA). At least 10,000 events from the target population (platelets or platelet EVs) were collected to ensure the statistical reliability of the results. The fluorescence of mNG-lactC2 was registered in the FITC channel (excitation: 488 nm; band filter: 530/30 nm), the fluorescence of TagBFP-lactC2 was registered in the Pacific Blue channel (excitation: 405 nm; band filter: 445/45 nm), and the fluorescence of AF647-labeled proteins was registered in the APC channel (excitation: 640 nm; band filter: 675/30 nm). All assays were performed in triplicate.

### 2.8. Thrombin Generation Assay

The kinetics of thrombin (coagulation factor IIa) generation in plasma was monitored by measuring the hydrolysis rate of the fluorogenic substrate Z-Gly-Gly-Arg-AMC (Bachem, Bubendorf, Switzerland) by thrombin formed during clotting, as described in [34], with minor modifications. Briefly, whole blood samples were collected from five healthy volunteers into tubes containing 3.8% sodium citrate. The samples were sequentially centrifuged at 1600× *g* for 15 min and then at 10,000× *g* for 5 min. The supernatant (platelet-free plasma, PFP) was collected, pooled, and stored at −80 °C until use. Prior to the experiment, the pooled PFP was thawed in a water bath at 37 °C for 15 min.

PFP (80 µL/well) was placed in the wells of a 96-well flat-bottom microtiter plate along with 10 µL of lactC2 and incubated for 30 min at RT. Subsequently, 10 µL of tissue factor (HemosIL RecombiPlasTin, Werfen, Boston, MA, USA) was added, and clotting was initiated by adding 20 µL of an activator consisting of the fluorogenic substrate mixed with CaCl_2_. The final concentrations of reagents in each sample were as follows: substrate, 400 µM; tissue factor, 5 pM; CaCl_2_, 16 mM; lactC2, 0–1000 nM. The activator was added to all wells simultaneously.

The kinetics of the accumulation of the fluorescent reaction product, 7-amino-4-methylcoumarin (AMC), was continuously monitored for 90 min at 37 °C using a PlateReader AF2200 (Eppendorf, Wesseling, Germany) at an excitation wavelength (λex) of 360 nm and an emission wavelength (λem) of 465 nm. All results represent the averages of three parallel measurements. A calibration curve was constructed by measuring the fluorescence of free AMC (Sigma Aldrich, MO, USA) in PFP (0–400 µM).

The rate of product accumulation is proportional to the instantaneous thrombin concentration in plasma. Therefore, the experimental curves of AMC accumulation were numerically differentiated to evaluate the thrombin generation curves. The contribution of the α2–macroglobulin–thrombin complex to the total thrombin activity was accounted for mathematically, as described in [34]. The following parameters of the thrombin generation test were measured in each case: endogenous thrombin potential (ETP), amplitude of the thrombin peak (Amax), time to reach the thrombin peak (Tmax), and time to produce 10 nM of thrombin (Tlag). A data analysis was performed using Origin 2018 software (OriginLab Corporation, Northampton, MA, USA).

## 3. Results

### 3.1. The Production of Soluble LactC2 in E. coli Is Enhanced by Attaching Soluble Fluorescent Proteins to the N-Terminus

**Figure 1:**
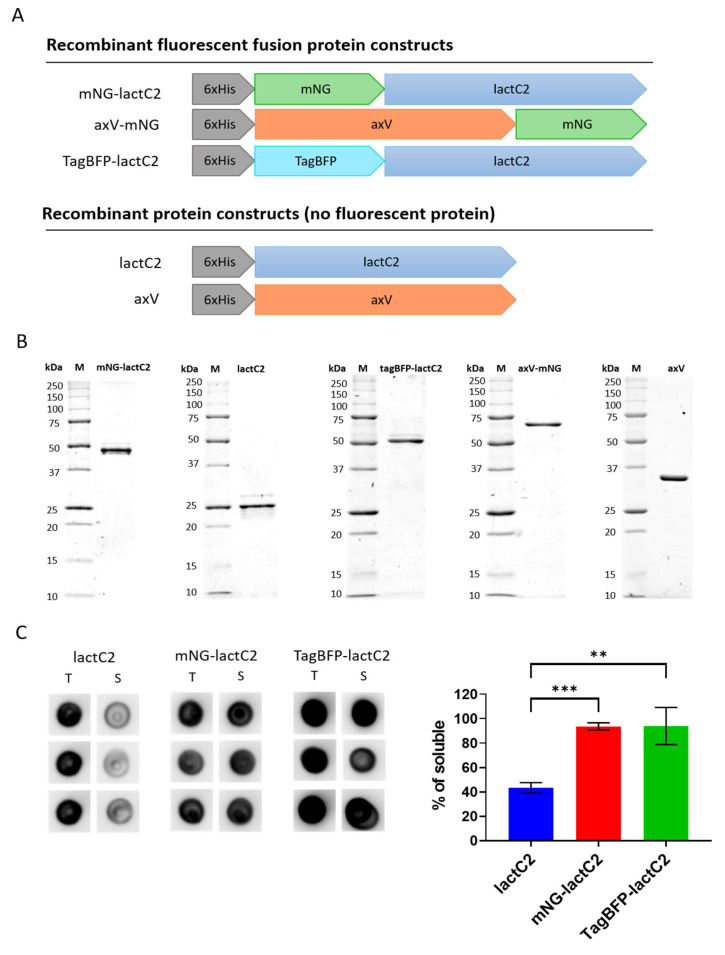
Recombinant protein PS sensors used in the current study. (**A**) Schematic representation of protein constructs. All proteins were N-terminally 6 × His-tagged. LactC2-based sensors contain the coding sequence of the bovine lactadherin C2 domain (residues 270–427). AxV-based sensors contain the coding sequence of human annexin V. (**B**) Coomassie Blue-stained SDS-PAGE gels of buffer-exchanged recombinant proteins. A total of 600 ng of protein was loaded onto a 12% SDS-PAGE gel. “M” indicates the protein molecular weight marker. Imaging was performed using a ChemiDoc MP Imaging system with Epi Far-Red illumination (Bio-Rad, Hercules, CA, USA). (**C**) A dot blot analysis of the lactC2-based sensors’ solubility. Three representative spots for the total fraction (T) and soluble fraction (S) for each protein are shown on the left. The dot blot was performed and analyzed as described in Section 2. The percentage of protein yield in the soluble fraction is shown on the right and was calculated as the ratio of the signal from the clarified lysate spots (soluble fraction) to the averaged signal from the crude lysate spots (total fraction). Data are represented as mean ± SEM. A statistical analysis was performed using Student’s t-test (** *p* < 0.01, *** *p* < 0.001).

A schematic representation of the protein constructs is shown in Figure 1A. The yields of the recombinant mNG-lactC2, TagBFP-lactC2, mNG-lactC2 mut, axV-mNG, lactC2, and axV were 5.1 mg, 2.5 mg, 6.5 mg, 3.4 mg, 0.7 mg, and 3.8 mg per 1 g of dry bacterial pellet mass, respectively. The purity of the proteins was >90%, as assessed using Coomassie Blue-stained SDS-PAGE gels of the final protein solutions (shown in Figure 1B). Since the final yield of the recombinant lactC2 was significantly lower compared to the variants carrying a fluorescent fusion tag, we compared the protein yields in the soluble fraction for all lactC2-based sensors used in the current study via dot blot assay. The yield of protein in the soluble fraction was approximately two times lower for lactC2 compared to the variants carrying a fluorescent fusion tag (Figure 1C). Raw images of the protein SDS-PAGE gels and a dot blot are presented in the Supplementary Materials.

### 3.2. Engineered Fluorescent LactC2 PS Sensors Are Comparable to Well-Characterized Annexin V-Based Probes in Flow Cytometry Experiments

**Figure 2:**
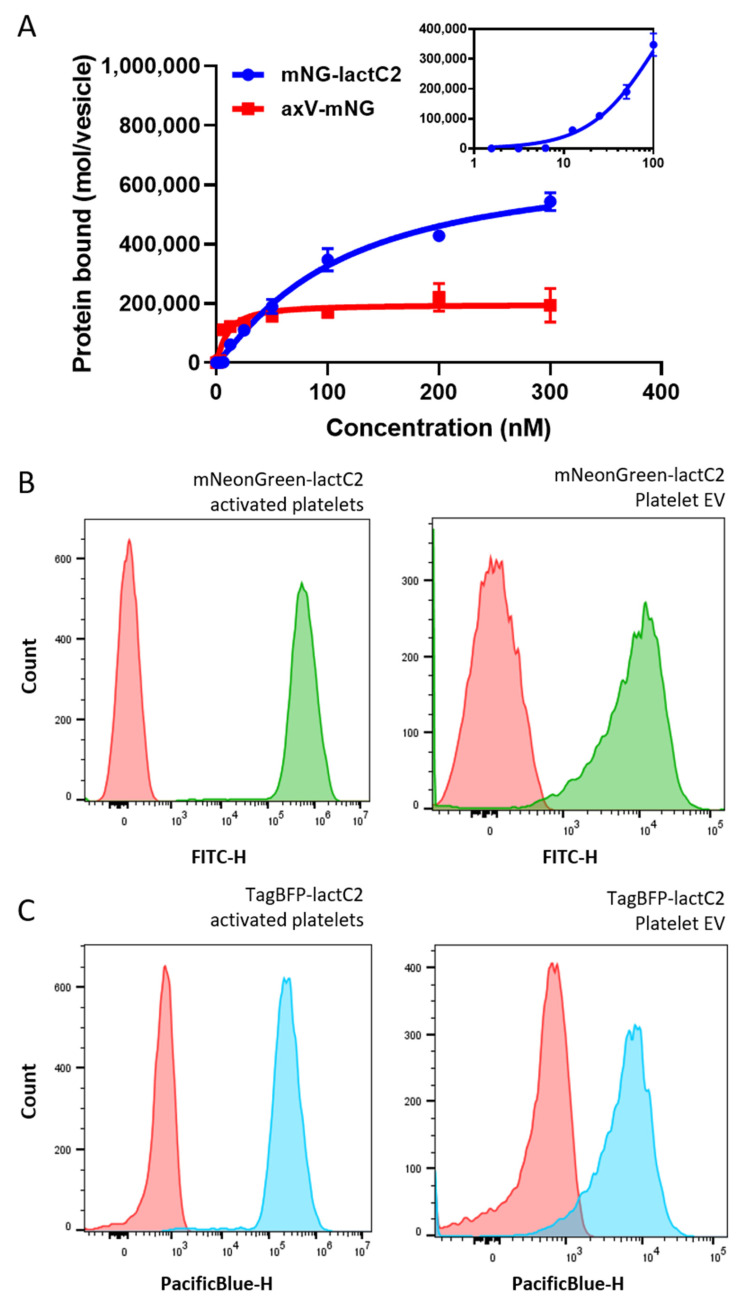
Binding of lactC2-based fluorescent PS sensors to various types of model membranes. (**A**) Equilibrium binding of mNG-lactC2 and mNG-axV to 20:80 PS/PC liposomes pre-stained with the DiD lipophilic dye. The mean binding parameters were calculated from four independent experiments. The number of bound protein molecules per liposome was estimated as described in Section 2. After the subtraction of non-specific binding, the binding curves were fitted with the Hill equation. Data are represented as mean ± SEM. The initial part of the mNG-lactC2 binding curve in logarithmic coordinates is shown as an inset. (**B**) Binding of 100 nM mNG-lactC2 to activated platelets (left) and platelet-derived extracellular vesicles (EVs) (right) (representative experiment histograms). Activated platelets and platelet EVs were obtained as described in Section 2. Platelet EVs were labeled with an anti-integrin IIb antibody (CD41-APC, Elabscience, China) followed by incubation with mNG-lactC2 for 30 min at RT. (**C**) Binding of 100 nM TagBFP-lactC2 to activated platelets (left) and platelet-derived extracellular vesicles (EVs) (right) (representative experiment histograms). Washed platelets were diluted in HBS containing 2.5 mM of calcium chloride and activated with 1 μM of calcium ionophore A23187 (Sigma Aldrich, MO, USA) for 10 min at RT. Activated platelets and platelet EVs were obtained as described in Section 2. Platelet EVs were labeled with an anti-integrin IIb antibody (CD41-APC, Elabscience, China) followed by incubation with TagBFP-lactC2 for 30 min at RT.

The fluorescent lactC2-based sensor containing mNG exhibited Ca^2+^-independent binding to both liposomes containing 20% PS (Figure 2A) and to activated platelets and platelet EVs (Figure 2B).

The fractional occupancy of the binding sites (*Θ*) was calculated as:

$$\Theta =\;\frac{B}{Bmax}$$

where *B* is the specific binding observed at a given ligand (protein) concentration (units: mol/liposome) and *B_max_* is the maximum specific binding derived from the experimental binding curve (units: mol/liposome).

Thus, the final version of the binding equation was:

$$B=\frac{Bmax\times {\left[L\right]}^{h}}{K_{d}^{h}+{\left[L\right]}^{h}}$$

where *B* is the specific binding observed at a given mNG-lactC2 or axV-mNG concentration (units: mol/liposome), *B_max_* is the maximum specific binding derived from the experimental binding curve (units: mol/liposome), [*L*] is the total mNG-lactC2 or axV-mNG concentration, *K_d_* is the mNG-lactC2 or axV-mNG concentration producing half occupation (microscopic dissociation constant), and *h* is the Hill coefficient.

Both sensors were best described by a sigmoidal binding curve with a Hill coefficient of ~1.2, suggesting potential cooperativity in lactC2 binding (the initial phase of the mNG-lactC2 binding curve in logarithmic coordinates is shown in the inset of Figure 2A). The apparent affinity (*K_d_*) of mNG-lactC2 was 107 nM, whereas mNG-axV exhibited a *K_d_* of 10 nM for the same vesicle type. The goodness of fit was assessed via the adjusted coefficient of determination (adjusted R^2^ = 0.98 for mNG-lactC2; 0.97 for axV-mNG). A residual analysis confirmed the random distribution of errors.

The staining of platelets activated with a calcium ionophore and platelet EVs with mNG-lactC2 resulted in a single-peak distribution of the FITC-positive population (Figure 2B), which was clearly distinguishable from the unstained control. Replacing the mNG fluorophore with TagBFP allowed the development of a stable and bright alternative sensor for the detection of PS on activated platelets and platelet EVs (Figure 2C).

### 3.3. NHS Ester-Based Labeling of LactC2 Significantly Impairs Protein Function

**Figure 3:**
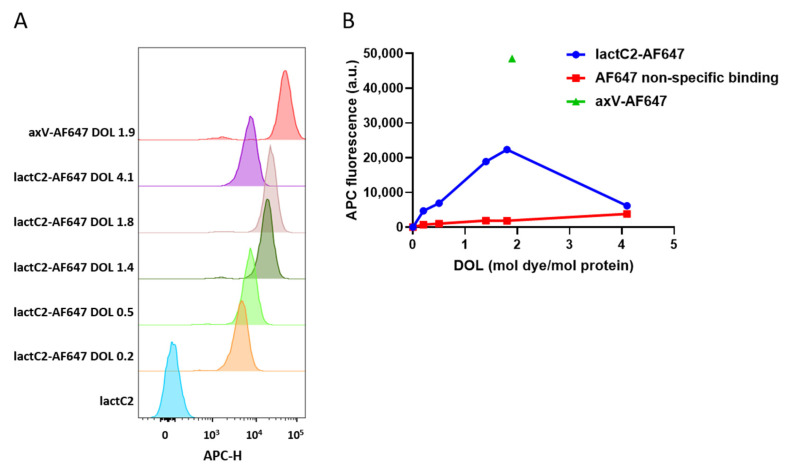
Binding of AF647-labeled lactC2 and annexin V (axV) to activated platelets. (**A**) Binding of lactC2-AF647 (degree of labeling (DOL): 0.2, 0.5, 1.4, 1.8, 4.1) compared to axV-AF647 (DOL 1.9) (representative experiment histograms). A concentration of 100 nM of lactC2-AF647 with varying DOLs or axV-AF647 was incubated with activated platelets for 30 min at room temperature (RT). (**B**) Dependence of mean APC fluorescence of lactC2-AF647 bound to activated platelets on DOL. To account for the non-specific background signal in the flow cytometry experiments, a free AF647 NHS ester was quenched with 50 mM of glycine. Free AF647 was added to the control wells at concentrations equivalent to those determined for labeled lactC2-AF647.

As the currently available far-red-emitting fluorescent proteins exhibit insufficient brightness for our experimental requirements, we opted to employ another strategy. Primary amine conjugation is one of the most robust and widely validated protein labeling approaches and it has been successfully implemented in previous studies using fluorescently labeled full-length lactadherin without reported functional impairment [35]. Therefore, we labeled recombinant lactC2 with AF647 via NHS chemistry. The labeling was successful; however, the resulting molecule was not as bright as axV labeled with the same AF647 NHS ester (Figure 3). The attempt to increase the DOL led to a decrease in the mean fluorescence of the 100 nM of lactC2-AF647 bound to the membranes of activated platelets (Figure 3A), as measured via flow cytometry. The dependence of the mean fluorescence of the bound lactC2-AF647 on the DOL exhibited a bell-shaped curve (Figure 3B), with values for DOL > 2 being approximately equal to the non-specific background.

### 3.4. LactC2 Inhibits Thrombin Generation in Platelet-Free Plasma but Only at High Concentrations

**Figure 4:**
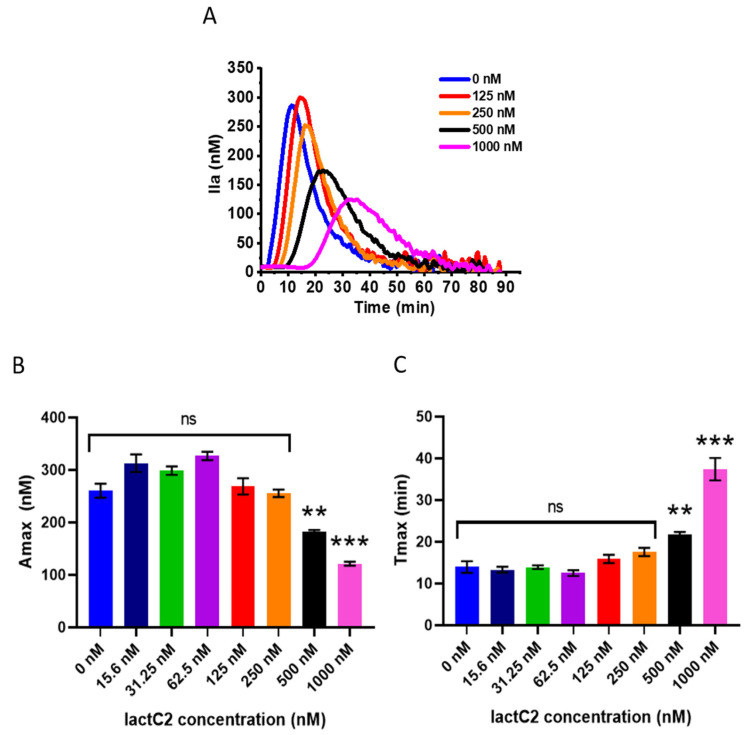
Inhibition of thrombin generation in platelet-free plasma by lactC2. (**A**) Thrombin generation curves in the presence of 0–1000 nM lactC2 (representative experiment). The kinetics of the accumulation of the fluorescent reaction product (AMC) was continuously monitored for 90 min at 37 °C, and the thrombin concentration at each time point was calculated as described in Section 2. Since the curves for 0–62.5 nM lactC2 completely overlapped, only the curves for 0 nM and 125–1000 nM concentrations are shown on the plot. (**B**) Maximal thrombin concentration (Amax) in the presence of 0–1000 nM lactC2. (**C**) Time to reach maximal thrombin concentration (Tmax) in the presence of 0–1000 nM lactC2. Data are represented as mean ± SEM. A statistical analysis was performed using a one-way ANOVA with post hoc Tukey’s multiple comparisons test (** *p* < 0.01, *** *p* < 0.001).

We performed a thrombin generation assay in the presence of increasing concentrations of lactC2 to determine whether competition for clotting factor–binding sites on native phospholipid vesicles present in plasma would translate into the inhibition of thrombin generation and whether the effect would be similar to that of full-length lactadherin. The results showed that no inhibition of thrombin production was observed at lactC2 concentrations below 250 nM (Figure 4A). Neither the parameters characterizing the total thrombin generated during the coagulation process (Amax (Figure 4B), ETP) nor those characterizing the rate of thrombin production (Tlag, Tmax (Figure 4C)) were altered at concentrations below 250 nM. However, at higher concentrations, the addition of lactC2 led to progressively longer inhibition of thrombin generation and a decrease in the total thrombin produced.

## 4. Discussion

The experimental results presented in this study provide valuable insights into the binding properties and functional implications of the lactC2-based fluorescent sensors for detecting PS on membranes. The findings highlight the unique characteristics of lactC2 compared to axV, a well-established PS-binding protein, and shed light on its utility as a fluorescent sensor for studying PS exposure in biological systems.

First and foremost, it is important to note that the tendency of full-length lactadherin and its isolated C2 domain to form aggregates is well-documented [36]. The C2 domain of lactadherin contains a 50 amino acid sequence (245–294), known as medin, which is responsible for this propensity [37]. Additionally, the literature highlights challenges in obtaining soluble protein in standard *E. coli* strains, with the exception of ArcticExpress(DE3), likely due to recombinant protein misfolding [21]. The conditions for recombinant synthesis in *E. coli*, as well as the protein isolation, purification, and storage methods described in the article, enable the production of soluble protein in *E. coli* with high yield and purity rates, while preventing the formation of insoluble protein aggregates during freezing and storage. It is also worth noting that the attachment of soluble fluorescent proteins to the N-terminus appears to increase the protein solubility, thereby increasing the yield of protein in the soluble fraction.

The lactC2-based sensors demonstrated Ca^2+^-independent binding to PS-containing liposomes, activated platelets, and platelet EVs. The microscopic dissociation constant (*K_d_*) of mNG-lactC2 for liposomes containing 20% PS was 107 nM, which is higher than that of mNG-axV (*K_d_* = 10 nM), indicating the lower affinity of lactC2 compared to axV in the chosen experimental model. However, the affinity of lactC2 for vesicles containing 20% PS determined in this study is higher than previously reported for similar experimental systems [23,38,39]. Most studies report that lactadherin binding to PS-containing membranes can be fitted to a one-site model [10,40,41]. However, the slightly sigmoidal binding curve observed in the current study for lactC2 suggests multistate binding, which is supported by both molecular dynamics simulations [42] and some previously reported experimental data [43].

The successful development of mNG-lactC2 and its TagBFP variant as bright and stable sensors for PS detection expands the toolbox for studying PS exposure in cells and EVs. The single-peak distribution of FITC- and Pacific Blue-positive populations in activated platelets and EVs stained with lactC2-based fluorescent probes indicates uniform and specific labeling, making it a reliable tool for flow cytometry-based assays. However, it is worth noting that despite the convenience in terms of production and the lack of need for optimization of the labeling conditions (since each functional molecule is always associated with one fluorophore), the fusion of the C2 domain with fluorescent proteins has significant limitations. Specifically, the spectrum of bright monomeric fluorescent proteins suitable for flow cytometry is highly restricted. The ability to label the functional molecule with low-molecular-weight dyes such as AF647 would help in overcoming these limitations. Unfortunately, the attempt to label lactC2 with the AF647 NHS ester revealed decreased brightness of the developed molecule compared to axV-AF647 at a comparable DOL, which is surprising given the higher number of lactC2 binding sites on the membrane, as demonstrated in experiments with model liposomes. The bell-shaped dependence of the fluorescence on the DOL suggests that this type of labeling may impair the binding efficiency of lactC2, possibly due to the modification of positively charged K24 and K45 residues, which have been reported to be involved in driving the docking of the PS head group into the loop regions of lactC2 [24]. Alternative approaches could include utilizing the HaloTag system or similar self-labeling platforms [44]. These fusion variants would enable conjugation with diverse fluorescent probes and ligands, potentially expanding the applications of our findings. However, as noted in our current study, such implementations would require careful evaluation of HaloTag’s effects on lactC2 domain solubility and function prior to adoption.

The thrombin generation assay revealed that lactC2 does not inhibit thrombin production at concentrations below 250 nM. However, at higher concentrations (>250 nM), lactC2 progressively inhibited thrombin generation, as evidenced by the prolonged inhibition and reduced total thrombin production. This concentration-dependent effect confirms that lactC2 indeed can interfere with the assembly of coagulation complexes on PS-rich membranes, similarly to full-length lactadherin [10,15,40,45,46], although only when present in excess. The lack of inhibition at lower concentrations distinguishes lactC2 from full-length lactadherin, which was reported to prolong the clotting time in whole blood already at concentrations of 40 nM [10]. It is possible that full-length protein provides more pronounced steric hindrance for the assembly of coagulation factor complexes on the membranes compared to the isolated C2 domain.

## 5. Conclusions

The findings of this study provide significant insights into the properties and potential applications of lactC2-based fluorescent sensors for detecting PS in biological systems. The development of recombinant mNG-lactC2 and its TagBFP variant as bright, stable, and Ca^2+^-independent sensors expands the toolkit for studying PS exposure in activated cells, apoptotic cells, and extracellular vesicles. However, we report significant detrimental effects of conjugation via primary amines, which limits the range of available fluorophores. LactC2 binds PS in PS-containing liposomes with a lower apparent affinity (*K_d_* = 107 nM) compared to axV. The slightly sigmoidal binding curve of lactC2 suggests multistate or cooperative binding.

In the context of coagulation, lactC2 does not inhibit thrombin generation at concentrations below 250 nM, distinguishing it from full-length lactadherin, which exhibits anticoagulant activity at much lower concentrations. This concentration-dependent effect limits its potential as a phospholipid-blocking anticoagulant but opens opportunities for its use as a sensor in experiments involving coagulation factors or in model systems where coagulation is required.

In summary, lactC2-based sensors offer a valuable addition to the tools available for studying PS exposure and membrane dynamics. While the utility of isolated lactC2 as a potential anticoagulant is limited, its Ca^2+^-independent binding, specificity, and adaptability for generating flow cytometry-compatible fluorescent labels make it a promising candidate for applications in cell biology, EV research, and coagulation studies. Future studies should focus on optimizing labeling strategies, exploring alternative fluorophores, and further elucidating the structural and functional mechanisms underlying lactC2’s unique binding properties.

## Data Availability

The data are contained within the article or Supplementary Materials.

## Supplementary Materials

The following supporting information can be downloaded at: https://www.mdpi.com/article/10.3390/biom15050673/s1, Table S1: Primers used for PCR of target fragments in current study; Figure S1: Dependence of binding of mNG-lactC2 and mNG-axV to 20:80 PS:PC liposomes pre-stained with the DiD lipophilic dye on incubation time; raw images of the protein SDS-PAGE gels and a dot blot.

## Abbreviations

Amax	Amplitude of the thrombin peak
AMC	7-Amino-4-methylcoumarin
APC	Allophycocyanin
axV	Annexin V
DOL	Degree of labeling
EDTA	Ethylenediaminetetraacetic acid
ETP	Endogenous thrombin potential
EVs	Extracellular vesicles
FITC	Fluorescein isothiocyanate
FSC	Forward scatter
HBS	HEPES-buffered saline
HRP	Horseradish peroxidase
IPTG	Isopropyl β-D-1-thiogalactopyranoside
lactC2	Lactadherin C2 domain
LB	Luria–Bertani (medium)
mNG	mNeonGreen
NHS	N-Hydroxysuccinimide
OD600	Optical density at 600 nm
PC	Phosphatidylcholine
PFP	Platelet-free plasma
PRP	Platelet-rich plasma
PS	Phosphatidylserine
RT	Room temperature
SDS PAGE	Sodium dodecyl sulfate–polyacrylamide gel electrophoresis
SSC	Side scatter
Tlag	Time to produce 10 nM of thrombin
Tmax	Time to reach the thrombin peak
