# Supplementary material for: Engineered Fluorescent Variants of Lactadherin C2 Domain for Phosphatidylserine Detection in Flow Cytometry
Source: Biomolecules. 2025 May 6;15(5):673. doi: 10.3390/biom15050673 (PMC12109337; doi:10.3390/biom15050673)
Supplement: Supplementary file 1 [file biomolecules-15-00673-s001.zip › biomolecules-3582386-supplementary.pdf]

Table S1. Primers used for PCR of target fragments in current study

Primer Name	Sequence (5′ to 3′)	Insert	Vector	
**pET-28a(+)-mNG-lactC2**				
Insertion of lactC2 into pmNeonGreenHO-G				
LactC2-F (1)	CAGCGT**AAGCTT**TATGCACTGAACCCCTAGGCC	LactC2	pmNeonGreenHO-G	Restriction-ligation
LactC2-R (2)	TGCACT**GTCGAC**ACAGCCCAGCAGCTCC			
Insertion of mNG-lactC2 into pet28a(+)				
mNG-LactC2-F	ACTTCC**GAATTC**ATGGTCAGCAAAGGCGAAGAAG	mNG-lactC2	pet28a(+)	Restriction-ligation
mNG-LactC2-R	TGCACT**GTCGAC**ACAGCCCAGCAGCTCC			
**pET-28a(+)-lactC2**				
LactC2-F (2)	GTAG**GAATTC**GCTTTATGCACTGAACCC	LactC2	pet28a(+)	Restriction-ligation
LactC2-R (2)	TTCCTTTCGGGCTTTGTTA			
**pET-28a(+)-TagBFP-lactC2**				
TagBFP-F	**TCGCGGATCCGAATTC**ATGAGCGAGCTGATTAAGGAG	TagBFP	pET-28a(+)-mNG-lactC2 (changing mNG to TagBFP)	Homologous recombination
TagBFP-R	**CTGAACCGCCTCCACC**ATTAAGCTTGTGCCCCAG			
pET-28a(+)-mNG-lactC2-F	GGTGGAGGCGGTTCA			
pET-28a(+)-mNG-lactC2-R	GAATTCGGATCCGCGACC			
**pET-28a(+)-axV**				
axV-F	TATACT**GGATCC**ATGGCACAGGT	axV	pet28a(+)	Restriction-ligation
axV-F	AGTCTG**GAATTC**TTAGTCATCTT			
**pET-28a(+)-axV-mNG**				
mNG-F	**CTGTGGAGAAGATGAC**GGAGGTGGCTCTATGGTCAGCAAAGGCG	mNG	pET-28a(+)-axV	Homologous recombination
mNG-R	**GCACTAATCCAGAAGA**TTACTTATAGAGTTCGTCCATGC			
pET-28a(+)-axV-F	TCTTCTGGATTAGTGCCAAG			
pET-28a(+)-axV-R	GTCATCTTCTCCACAGAGC			
**pET-28a(+)-mNG-lactC2 mut W26A/G27A/L28A**				
mNG-lactC2 mut1-F	*GCGGCAGCTAGTGCCTTTAGCTGGTTTC	-	pET-28a(+)-mNG-lactC2	Circularization of PCR-product with T4 ligase
mNG-lactC2 mut1-R	*GGTTTTGTAGTAGCTGGAGG			
**pET-28a(+)-mNG-lactC2 mut W26A/G27A/L28A/F81A/G82A**				
mNG-lactC2 mut2-F	*GCCGCACACATTCAATATGTGGCTGCCTAC	-	pET-28a(+)-mNG-lactC2 mut W26A/G27A/L28A	Circularization of PCR-product with T4 ligase
mNG-lactC2 mut2-R	*GTCTCGGGCACCCTG			
**Primers for Sanger sequencing**				
T7 promoter	TAATACGACTCACTATAGGG	-	-	Sanger sequencing
T7 terminal	GCTAGTTATTGCTCAGCGG			

*5′-phosphorylated primers

For mutagenesis primers, modified bases are underlined; letters in **bold** denote either introduced restriction sites or homology arms

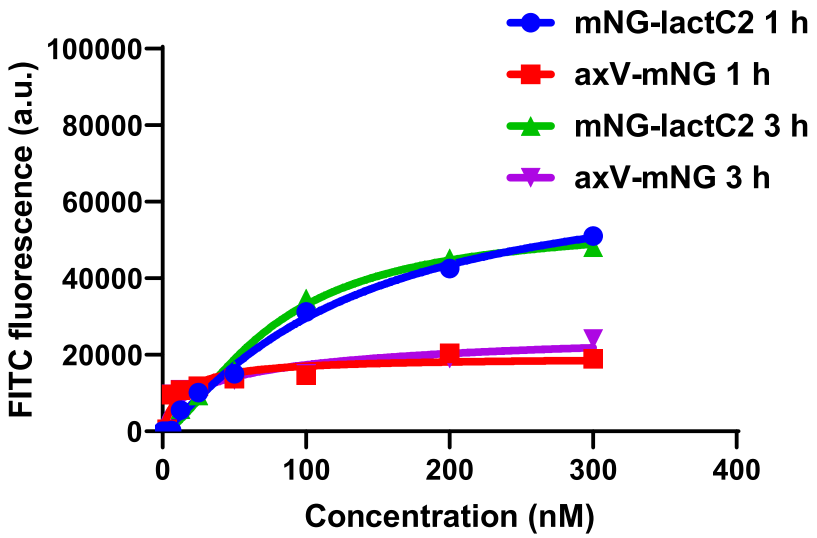

Figure S1. Dependence of binding of mNG-lactC2 and mNG-axV to 20:80 PS:PC liposomes pre-stained with the DiD lipophilic dye on incubation time. Liposomes were incubated for 1 hour with various concentrations of mNG-lactC2 in HBS or mNG-axV in HBS containing 2.5 mM $CaCl_2$. Liposomes labeled with mNG-lactC2 mut were used as a specificity control for mNG-lactC2 binding. Liposomes labeled with mNG-axV in the presence of 10 mM EDTA were used as a specificity control for mNG-axV binding. Measurement was repeated after 3 hours of incubation in the same wells. After subtraction of non-specific binding, the binding curves were fitted with the Hill equation.
