# Supplementary figures and images for: Engineered Fluorescent Variants of Lactadherin C2 Domain for Phosphatidylserine Detection in Flow Cytometry
Source: Biomolecules. 2025 May 6;15(5):673. doi: 10.3390/biom15050673 (PMC12109337; doi:10.3390/biom15050673)

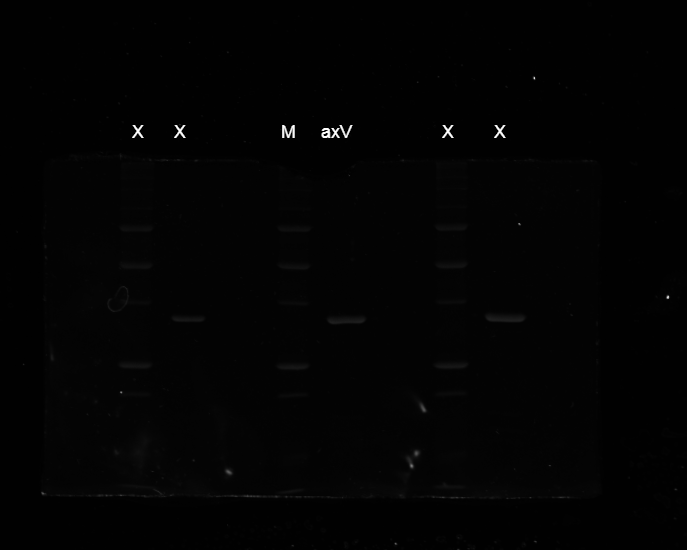

Supplement: Supplementary file 1 [file biomolecules-15-00673-s001.zip › axV (Coomassie Blue).raw16.png]

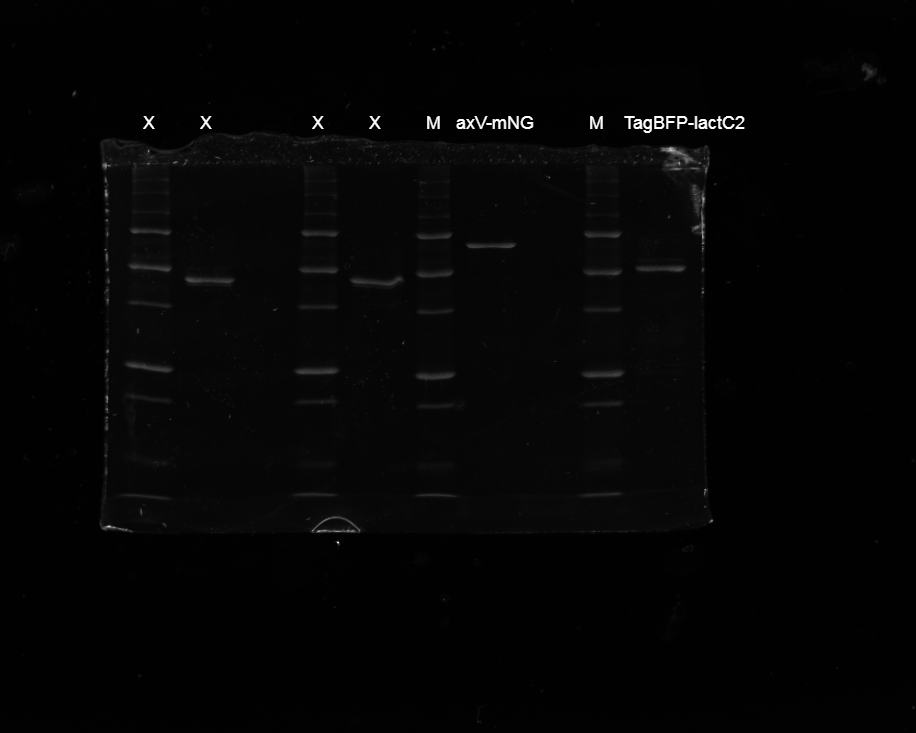

Supplement: Supplementary file 1 [file biomolecules-15-00673-s001.zip › axV-mNG and TagBFP-mNG (Coomassie Blue).raw16.png]

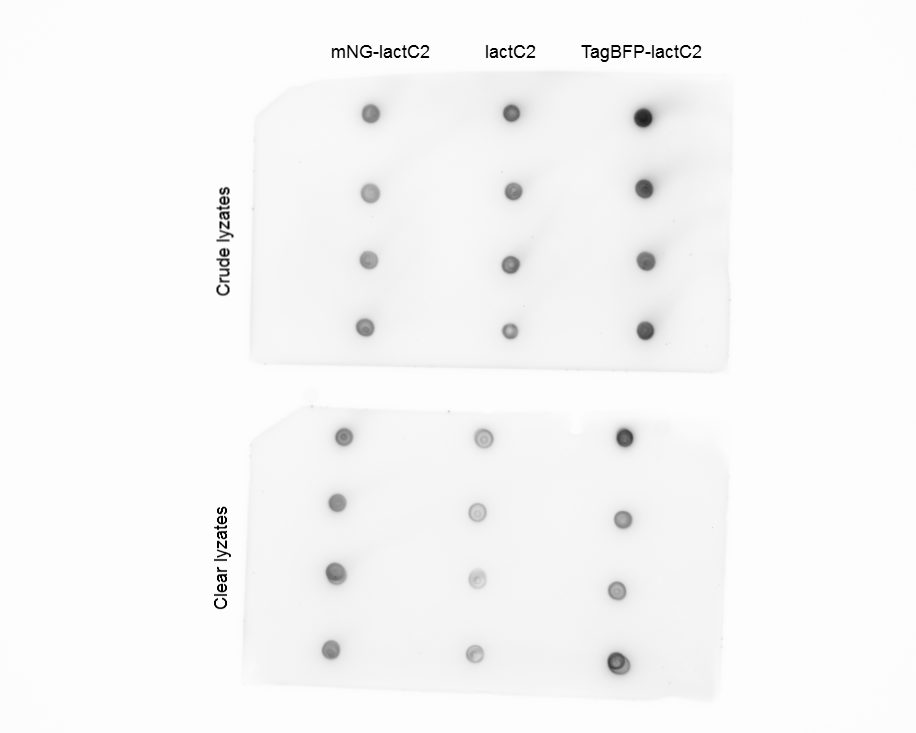

Supplement: Supplementary file 1 [file biomolecules-15-00673-s001.zip › dot blot raw (Chemiluminescence).raw16.png]

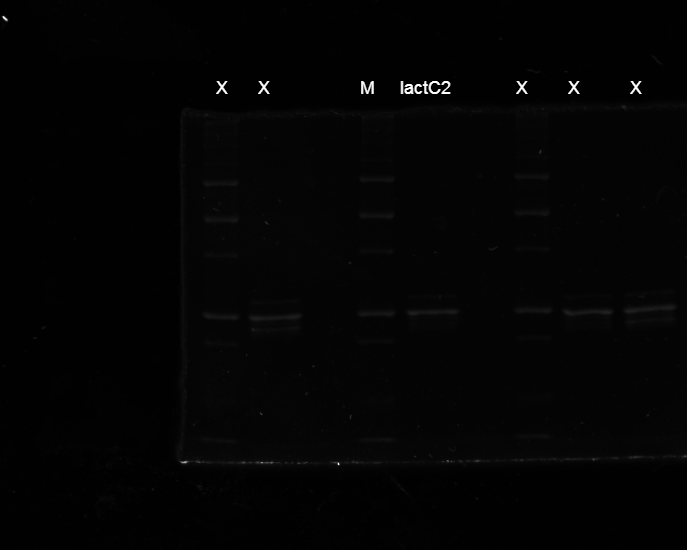

Supplement: Supplementary file 1 [file biomolecules-15-00673-s001.zip › lactC2 (Coomassie Blue).raw16.png]

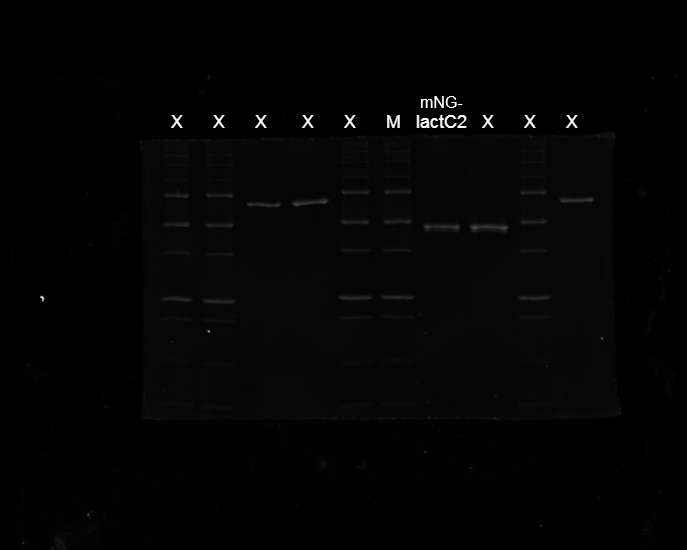

Supplement: Supplementary file 1 [file biomolecules-15-00673-s001.zip › mNG-lactc2 (Coomassie Blue).raw16.png]
